# Atypical cutaneous manifestations: When leprosy and syphilis overlap

**DOI:** 10.1590/0037-8682-0391-2025

**Published:** 2026-02-16

**Authors:** Natália Yasmin de Souza, Beatriz Zimermano Coimbra, Angela Marques Barbosa, Deusita Fernandes Gandia Soares, Marilda Aparecida Milanez Morgado de Abreu

**Affiliations:** 1 Hospital Regional de Presidente Prudente, Departamento de Dermatologia, Presidente Prudente, SP, Brasil.; 2 Universidade do Oeste Paulista, Programa de Pós-Graduação Stricto Sensu em Ciências da Saúde, Presidente Prudente, SP, Brasil.; 3 Universidade do Oeste Paulista, Faculdade de Medicina, Presidente Prudente, SP, Brasil.

**FIGURE 1:**
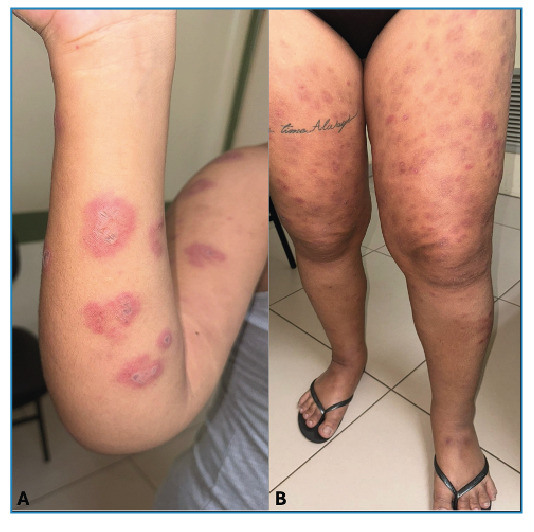
**(A)** Erythematous-edematous plaques and nodules with central crust on the right upper limb. **(B)** The same lesions on the lower limbs, along with hyperchromic macules.

**FIGURE 2:**
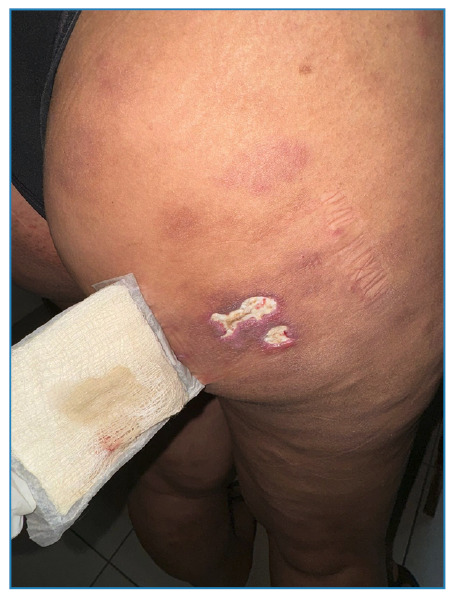
Two erythematous plaques with infiltrated borders and central ulceration with purulent exudate in the right gluteal region.

**FIGURE 3:**
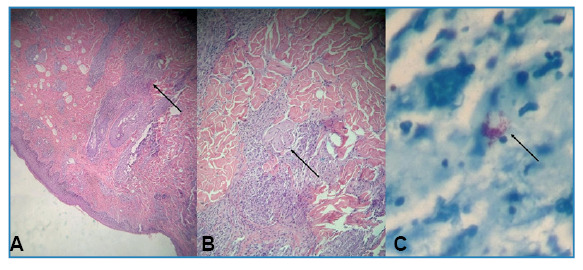
**(A)** Inflammatory process involving nerve bundles observed with hematoxylin-eosin staining (×40). **(B)** Higher magnification of the inflammatory process (×100). **(B)**Presence of globi with Ziehl-Neelsen staining (×400).

We report the case of a 23-year-old Brazilian woman with simultaneous syphilis and leprosy. Dermatological examination revealed erythematous nodules and plaques with central ulcerations on the upper and lower limbs (Figures 1 and 2). She described experiencing skin lesions for five months, initially starting as erythematous papules on her right thigh that later spread, accompanied by fever. She was previously diagnosed with syphilis (Venereal Disease Research Laboratory - VDRL ratio 1:16) and treated with benzathine penicillin (2,400,000 IU weekly for three weeks), resulting in partial improvement. Owing to residual pruritus, she received prednisone (40 mg/day for 7 d), which led to complete remission. Five days after medication discontinuation, the lesions recurred, along with fever and myalgia. When the VDRL ratio was increased to 1:32, Fluorescent Treponemal Antibody Absorption - FTA-Abs became reactive. Doxycycline (100 mg every 12 h) was prescribed; however, the lesions did not fully resolve. Biopsies of the two skin lesions showed findings consistent with borderline lepromatous leprosy (Figure 3). Multidrug therapy with rifampicin, dapsone, and clofazimine was initiated during outpatient follow-up. Leprosy is a chronic infectious disease caused by *Mycobacterium leprae* with an incubation period of 5-20 years^1^ ^-^ ^3^ . It mainly affects the peripheral nervous system and skin, and can resemble other skin conditions, such as syphilis. Syphilis is a chronic bacterial infection caused by *Treponema pallidum*, characterized by diverse skin symptoms and systemic signs that can also look like those of leprosy^1^ ^,^ ^2^. This case highlights the importance of skin biopsy in atypical clinical presentations and emphasizes the need to consider leprosy in the differential diagnosis of persistent skin lesions in endemic areas, particularly in cases of coinfection.
